# A novel IoT intrusion detection framework using Decisive Red Fox optimization and descriptive back propagated radial basis function models

**DOI:** 10.1038/s41598-024-51154-z

**Published:** 2024-01-03

**Authors:** Osama Bassam J. Rabie, Shitharth Selvarajan, Tawfiq Hasanin, Abdulrhman M. Alshareef, C. K. Yogesh, Mueen Uddin

**Affiliations:** 1 Department of Information Systems, Faculty of Computing and Information Technology, King Abdulaziz University, Jeddah, Kingdom of Saudi Arabia; 2 Cybersecurity Center, King Abdulaziz University, Jeddah, Kingdom of Saudi Arabia; 3 School of Built Environment, Engineering and Computing, Leeds Beckett University, Leeds, LS1 3HE UK; 4 Department of Computer Science, Kebri Dehar University, Kebri Dehar, Ethiopia; 5 School of Computer Science and Engineering, ViT Chennai Campus, Chennai, India; 6 College of Computing and IT, University of Doha for Science and Technology, 24449 Doha, Qatar

**Keywords:** Engineering, Electrical and electronic engineering

## Abstract

The Internet of Things (IoT) is extensively used in modern-day life, such as in smart homes, intelligent transportation, etc. However, the present security measures cannot fully protect the IoT due to its vulnerability to malicious assaults. Intrusion detection can protect IoT devices from the most harmful attacks as a security tool. Nevertheless, the time and detection efficiencies of conventional intrusion detection methods need to be more accurate. The main contribution of this paper is to develop a simple as well as intelligent security framework for protecting IoT from cyber-attacks. For this purpose, a combination of Decisive Red Fox (DRF) Optimization and Descriptive Back Propagated Radial Basis Function (DBRF) classification are developed in the proposed work. The novelty of this work is, a recently developed DRF optimization methodology incorporated with the machine learning algorithm is utilized for maximizing the security level of IoT systems. First, the data preprocessing and normalization operations are performed to generate the balanced IoT dataset for improving the detection accuracy of classification. Then, the DRF optimization algorithm is applied to optimally tune the features required for accurate intrusion detection and classification. It also supports increasing the training speed and reducing the error rate of the classifier. Moreover, the DBRF classification model is deployed to categorize the normal and attacking data flows using optimized features. Here, the proposed DRF-DBRF security model's performance is validated and tested using five different and popular IoT benchmarking datasets. Finally, the results are compared with the previous anomaly detection approaches by using various evaluation parameters.

## Introduction

Internet of Things (IoT) has recently drawn increased attention because of its innovative uses and support for various industries, including industrial applications, healthcare, transportation, ambient intelligence^1^, etc. IoT offers a vast range of applications and services but also confronts serious security risks and assaults. Since the IoT is a heterogeneous environment, traditional security techniques are not supported by its interoperability mechanism^2^. IoT security is improved in other ways, such as data authentication, secrecy, and access controls^3^. However, IoT networks are susceptible to numerous assaults that try to disrupt the web, even with these defenses. A separate module must therefore ensure the security of the IoT network. One such idea is the intrusion detection system (IDS)^4,5^, which is already utilized in wireless networks. Also, it helps to secure the network from assaults and other vulnerabilities by improving the IDS features of wireless networks. Specifically, the IDS^6–8^ is treated as the essential element in enhancing the cybersecurity of IoT networks, which is also highly suited for both fog and cloud platforms. Moreover, it uses the internet and real-time applications to offer users an efficient and convenient environment. Therefore, before deploying an IDS^9,10^, it is essential to analyze the security challenges in the network. Some of the significant properties used to ensure the security of IoT networks are as follows: data confidentiality, authentication, integrity, availability, and authorization^11^. The three primary functional mechanisms that most existing IDS^12–16^ use are as follows:

- *Sources of information* When determining if an intrusion has occurred, sources of information such as incoming packets or data are considered.
- *Characterization* The required method determines when the events gathered suggest that intrusions are happening or have already happened. The most popular analysis techniques are misuse detection and anomaly detection.
- *Reaction* When the system notices an intrusion, it sends a response. There are two types of reaction measures: active and passive. A functional response measure occurs when the system takes action on its own, whereas a passive response measure sends its findings to the administrator, who may take action based on these reports.

Also, various machine learning and deep learning^17,18^ based AI mechanisms are used in the traditional works for developing an effective IDS. Machine learning is a kind of artificial intelligence that systematically uses algorithms to discover the underlying connections between data and information. It is categorized into the types of supervised learning, unsupervised learning, and reinforced learning. Similarly, the deep learning techniques^19^ are also increasingly used nowadays, which is an extended version of machine learning. However, the conventional classification methodologies^4^ face the challenges associated to the factors of increased time consumption, overfitting, reduced processing speed, high false positives, and difficulty in understanding.

The IoT delivers innovative features and services to a large number of consumers, hence enhancing their lifestyles. Most IoT devices and objects don’t require a lot of capacity. The IoT has a limited amount of available storage and transmission capacity. As a result, clouds are used to store a vast amount of confidential documents. This increases the availability and accessibility of the services supplied while lowering the expenses and effort. This technology enables the users to access the applications and services at anywhere & anytime, which creates a significant challenges to the data security. Moreover, some other factors such as cost, performance, data scalability and availability are also considered as the IoT related challenges. Since, there is no standard format or protocol for the data transmission, storage, maintenance and etc in IoT, when it is dealing with vast amount of data. Some of these issues usually take the form of network anomalies, like a deviation from normal network action. The IoT devices are becoming more prevalent in today's world, yet the cloud has significant restrictions as listed below:

- More energy consumption
- Increased network bandwidth consumption
- High latency or delay
- Outage of internet
- High maintenance cost due to an unwanted data storage
- Minimal control over the applications or data
- Security breaches

Due to the IoT features such as flexible data sharing and constant connectivity, there are a number of cybersecurity problems have been created with this development. To resolve this problem, many IDS are developed for assuring IoT security, which showed their effectiveness in mitigating cyber-threats. Specifically, the deep learning algorithms are increasingly used in the existing works for enhancing the attack detection rate in an IoT networks. However, the existing deep learning techniques are highly complex to interpret, and their prediction decisions are very difficult to understand by the cybersecurity experts. As a result, the corresponding users are unable to both understand and trust the decisions made by DL models and to optimize their own actions in light of those decisions. Therefore, the proposed work motivates to develop an efficient and highly secured IDS framework for IoT security. The main purpose of this research article to design and develop a novel IoT based intrusion detection framework for maximizing the security with lower computational burden. It also intends to maintain an improved detection performance and results while accurately predicting the type of intrusion from the large/huge dimensional intrusion datasets. For accomplishing these objectives, the different kinds of mining techniques including preprocessing, DRF based feature selection, and DBRF based classification are implemented in this study.

The major research contributions of this paper are as follows:

- In order to generate a balanced dataset that will increase the detection rate and precision of IDS, data preprocessing is carried out, which includes handling of NaN values, the extraction of categorical features, and the identification of missing fields.
- A Decisive Red Fox (DRF) optimization approach is used to extract the pertinent features from the balanced IoT datasets, which improves the classifier's training process.
- The use of a Descriptive Back Propagated Radial Basis Function (DBRF) classification method allows the identification and categorization of intrusions in IoT systems based on the features of data.
- To validate and compare the results of proposed DRF-DBRF security framework, various evaluation indicators as well as the popular IoT IDS datasets are utilized in this work.

The remaining sections of this article are divided into the following categories: The traditional approaches to enhancing the security of IoT networks are reviewed in “Related works” section. Additionally, it verifies the benefits and drawbacks of each mechanism in light of the effectiveness and outcomes of its attack detection. The suggested DRF-DBRF methodology is fully explained in “Methods” section together with the overall work flow and algorithms. Additionally, “Results” section compares and validates the performance and outcomes of the proposed technique using a variety of performance indicators. Finally, “Conclusion” section summarizes the entire work together with the conclusions and future scope.

## Related works

The comprehensive literature review of the IDS frameworks currently in use for enhancing the security of IoT networks is presented in this part. Furthermore, it examines each model's benefits and drawbacks in context of its effectiveness and reliability in detection.

**Figure 1:**
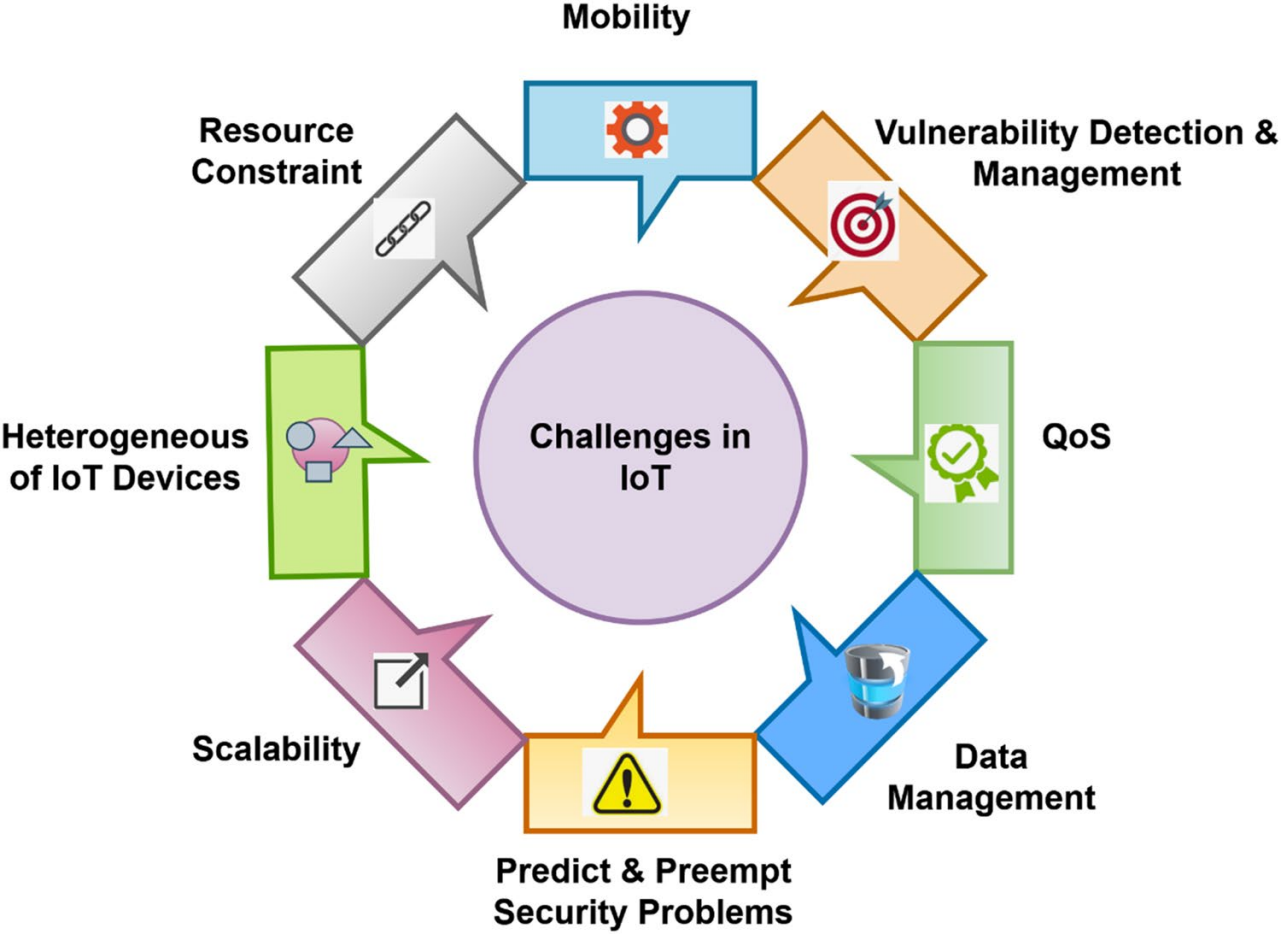
Challenges in IoT systems.

Gu et al.^20^ utilized a Convolutional Neural Network (CNN) mechanism for developing an accurate IDS framework to ensure the security of IoT networks. Here, the Kitsune network attack database has been utilized to implement this system, which comprises the different types of network attacks. The CNN has the ability to automatically recognize the data packets for ensuring a secured end-to-end communication in IoT systems. However, the CNN model requires a lot of training data to predict an accurate results, and it has a reduced learning speed. Alsoufi et al.^21^ presented a comprehensive literature review to examine various deep learning techniques for designing an effective anomaly detection system. Also, it intends to increase the detection accuracy, and minimize the false alarm rate by solving the security problems in the IoT networks. Here, the 11 different types of attack datasets have been utilized to validate the system model using various parameters. According to this survey, it is observed that developing a lightweight anomaly detection mechanism could be highly beneficial for the IoT systems. According to this study work, it is noted that the majority of deep learning mechanisms facing challenges in high computational complexity while training samples for classification, increased time consumption for both training and testing operations, and overfitting outcomes. Mishra et al.^22^ presented a comprehensive literature review to analyze the security challenges, vulnerabilities, and attacks in the IoT networks. The authors of this paper intend to conduct a multi-fold survey for analyzing the security issues in the IoT layers. Typically, ensuring the parameters such as interoperability, connectivity, and standardization were considered as the major security challenges of IoT networks, which is graphically represented in Fig. 1.

The main focus of this paper is to study the different types of DDoS attacks with their mitigation strategies. Here, the various types such as volumetric attack, protocol based attack, and application layer attack are discussed with the goal of attacker and the preventive solutions. As its name implies, a DDoS attack aims to overload a target and stop services from functioning. IoT devices are highly suited for the DDoS attack because it needs a lot of devices to initiate an attack. Also, the users will typically not be aware that a device is compromised. The suggested work only focused on detecting DDoS attacks from the network, since some of the modern attacks could degrade the performance of wireless networks in present days. Fatani et al.^23^ utilized an aquila optimization technique integrated with the deep learning mode for developing an efficient IDS for IoT systems. Here, the CNN algorithm was utilized for extracting the relevant features from the given attack datasets. Then, the binary aquila optimization algorithm was deployed for choosing the optimal features with increased classification accuracy. Finally, the ML classification algorithm was deployed to categorize the type of attacks according to the reduced features. However, the suggested optimization technique having the specific drawbacks of local optimum, lower searching efficiency, and increased time for finding optimal solutions.

Abd-Elaziz et al.^24^ developed a new capuchin search algorithm incorporated with the deep learning model for detecting intrusions from cloud-IoT systems. The purpose of this paper is to implement a new feature selection based deep learning algorithm for assuring the security of IoT systems. Here, various and recent Cloud-IoT datasets have been utilized to validate the performance of the suggested mechanism. The outcomes of this analysis depict that the suggested technique provides a competitive performance for all datasets utilized in this work. Nevertheless, the suggested deep learning algorithm requires lot of training samples to predict the accurate results. Aslam et al.^25^ introduced an adaptive machine learning based security methodology for protecting SDN from cyber-attacks. Here, an adaptive multi-layered feed forward mechanism is deployed to accurately spot the DDoS attacks by analyzing the features of the network traffic. Moreover, this framework provides an increased accuracy with low false alarm rate. But, it failed to focus some of the modern attacks or vulnerabilities that degrade the security of SDN. Smys et al.^26^ introduced a hybrid IDS for protecting IoT system against network vulnerabilities and harmful intrusions. The motive of this work was to guarantee the properties of data confidentiality, integrity, availability, authorization, and authentication for IoT security. Typically, the three different types of security schemes were used for IoT networks, which includes placement strategy, detection strategy, and validation strategy. In this work, the LSTM-RNN model was used to detect the network anomaly with improved performance. Moreover, this framework comprises the working stages of log file generation, feature extraction, encoring, matrix formation, classification, and intrusion categorization. However, the suggested methodology was not more suitable for handling the complex network datasets, which could be the major limitation of this work. Almiani et al.^27^ implemented a Deep Recurrent Neural Network (DRNN) for increasing the security of IoT networks. It encompasses the major operations of feature reduction, data normalization, over sampling, and intrusion detection. In the suggested framework, the common mining operations including sampling, normalization, feature elimination, and intrusion identification processes are performed. For classification, the DRNN technique is implemented here, which follows some complex mathematical models to accurately predict the type of intrusion. Hence, it may be difficult to understand the classification operations of the suggested technique. Verma et al.^28^ deployed an ensemble of machine learning classifiers for detecting intrusions from the IoT networks. It includes Random Forest (RF), Gradient Boosted Machine (GBM), Extreme Gradient Boost (EGB), Extremely Randomized Trees (ERT), Classification & Regression Trees (CART), and Multi-Layer Perceptron (MLP). Consequently, various benchmarking datasets have been used to validate the performance of these classifiers. Based on this investigation, it is identified that the CART outperforms the other machine learning models with improved attack detection accuracy. Yet, it follows some complex mathematical modeling for attack prediction and classification. Anthi et al.^29^ developed a three layered IDS framework using a supervised learning methodology for protecting IoT networks. This framework comprises the following operations:

- IoT device behavior analysis
- Malicious packet identification
- Attack class categorization

Specifically, the authors intend to design and develop a lightweight security framework for detecting cyber-attacks in the smart home IoT networks. The advantages of this framework were increased attack detection accuracy, better efficacy, easy deployment, and reduced overfitting. However, the time required for training and testing the features while classifying the type of data need to be reduced. Al-Hadhrami et al.^30^ introduced a real time dataset generation framework for spotting intrusions in the IoT networks. In this work, the problems and limitations associated to the existing IDS datasets have been discussed. Moreover, the key components involved in this framework were capturing medium, data aggregation, feature extraction, and queuing unit. Benkhelifa et al.^31^ presented a critical review to protect the IoT networks against the network intrusions. The purpose of this paper was to develop a highly secure and robust IDS framework for analyzing the malicious behavior of nodes. The different types of detection methodologies reviews in this work were anomaly detection models, specification based detection methods, and hybrid detection models. Qureshi et al.^32^ introduced a heuristic based detection mechanisms for protecting IoT networks, which includes the modules of data preprocessing, classifier training and testing. During dataset processing, the attribute selection, one hot encoding, and normalization operations were performed to improve the training and testing processes. Moreover, it accurately predict the normal and attacking data traffic flows based on the features training features. Due to the increased dimensionality of features, the overall attack detection accuracy and efficiency of classification have been affected. Kumar et al.^33^ introduced a Unified IDS framework for strengthening the security IoT networks against four different types of attacks such as exploit, DoS, probe and generic. Here, the dataset clustering was performed at the initial stage for analyzing the behavior of attacks. Then, the rule generation and integration operations were performed to extract the relevant features for classifier training and testing. This framework is not capable of handling huge datasets with low time and computational complexity.

This part presented the related works that review and outline intrusion detection strategies utilizing machine learning/deep learning algorithms in the IoT network by emphasizing their key contributions. In several studies, the topics of IoT security, privacy, and intrusion detection are addressed. Although several research studies^34^ on intrusion detection systems in IoT applications are still in the development phase. The study indicates that much of the existing research work faces several challenges while ensuring security in IoT. Hence, it is most important to resolve the following problems for developing an effective IDS: computational burden, increased amount of time for prediction, inability to handle a vast amount of data, and high false positives. As a result, the proposed study aims to create an intelligent and efficient IDS framework for enhancing IoT security against dangerous network intrusions.

## Methods

**Figure 2:**
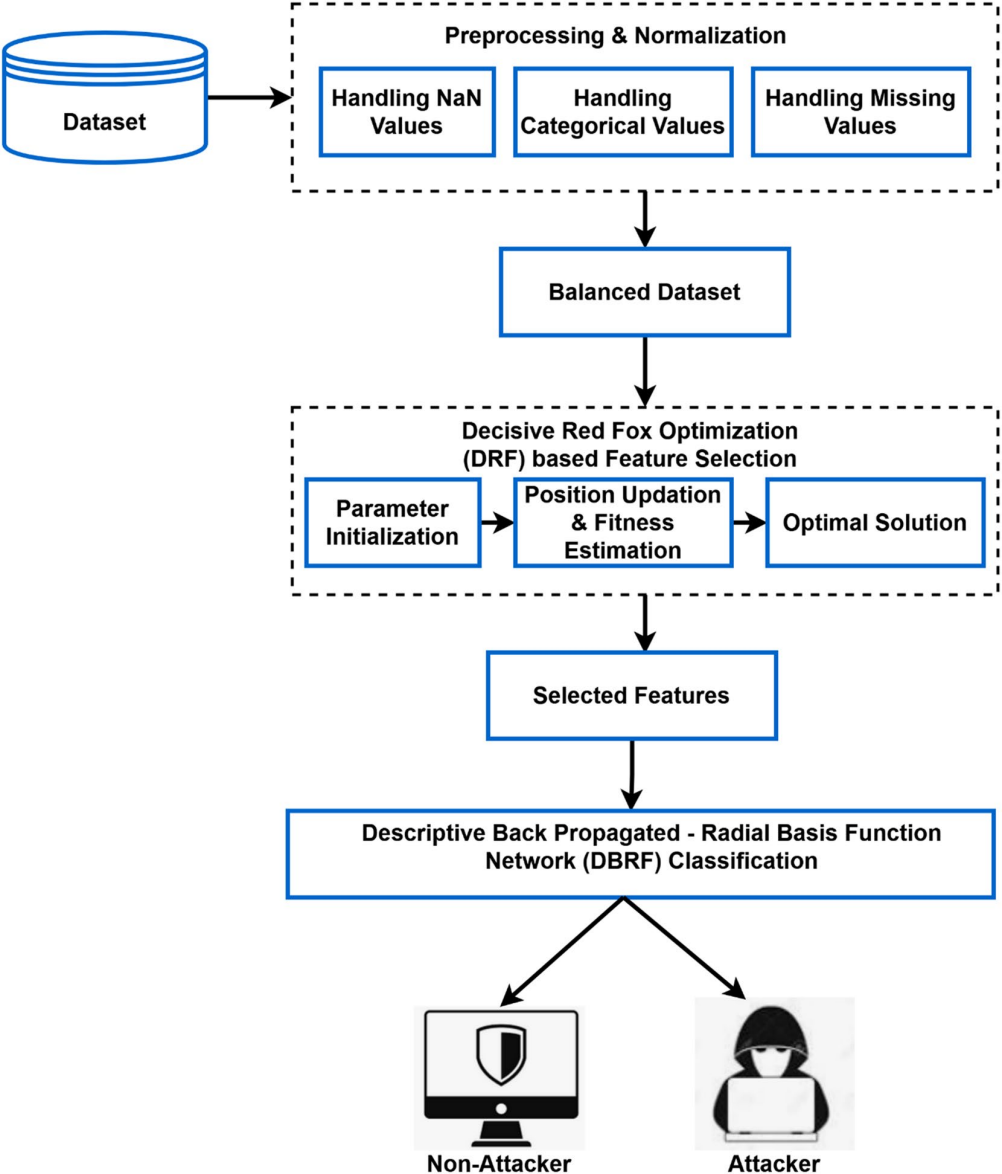
Workflow model of the proposed security framework.

This section provides the complete explanation for the proposed security model used to protect IoT systems. The IoT technologies are anticipated to provide a new level of communication with the use of smart devices, which can improve regular chores and enable smart decisions based on sensed data. The original contribution of the proposed work is to develop an intelligent IoT intrusion detection framework with the use of advanced DRF and DBRF techniques. By using the combination of these methodologies, the overall performance and efficacy of the intrusion detection system is greatly improved with high accuracy, lower training and testing time. Moreover, this eliminates the need of complex mathematical calculations for preprocessing, feature optimization, and classification operations. In order to determine its efficacy and superiority, the most recent and huge dimensional IoT intrusion datasets are taken into account for performance validation and assessment. The sensitive data collected by the IoT must be protected from assaults and privacy concerns. Moreover, the IoT security is a hotly debated topic in both academia and business in present days. In fact, attacks to IoT products and services could result in security breaches and information leakage. The purpose of this work is to design an IDS framework using machine learning technique, with the goal of detecting attempts to exploit IoT systems and to mitigate hostile occurrences. The original contribution of this work is to develop a highly efficient and accurate IDS framework for securing the IoT networks by using a novel data mining methodologies. For accomplishing this objective, a novel Decisive Red Fox optimization (DRF) and Descriptive Back Propagated-Radial Basis Function (DBRF) network classification models are deployed, which helps to strengthen the security of IoT networks. The overall work flow of the proposed system is shown in Fig. 2, which comprises the following operations:

- Data preprocessing & normalization
- Decisive Red Fox (DRF) optimization based feature selection
- Descriptive Back Propagated-Radial Basis Function (DBRF) network based classification
- Attack identification and categorization
- Performance evaluation

Here, the popular IoT IDS datasets such as IoTID-20, NetFlow-BoT-IoT-v2, NF-ToN-IoT-v2, NSL-KDD, UNSW-NB 15 datasets have been used for system implementation. The raw network datasets are noisy, which holds some irrelevant attributes, and missing fields. As a result, it affects intrusion detection and classification performance and outcomes. Thus, the data normalization and preprocessing operations are performed in this framework, which holds the operations of handling Not a Number (NaN) values, handling categorical values, and missing values. In the proposed work, the imbalanced dataset is handled by using the random over-sampler to preprocess the incoming data, handling missing values, categorical features, NaN values, and unbalanced datasets. Data cleansing, visualization, feature engineering, and vectorization are typically done as part of the dataset preprocessing procedure. To extract data from the data collection, all of these methods have been applied. Two sets of these characteristic vectors have been generated, one for training and the other for testing, with 80:20 proportion between the two sets. An unbalanced dataset, missing values, categorical features, and NaN value handling are the four processes used in the proposed work to deal with the incoming data. Here, the NaN value handling is mainly performed to highly increase the accuracy of intrusion recognition and classification. After successfully handling NaN values, the next step in handling categorical features is processing those characteristics. This stage involves handling categorical data before it is fed into artificial intelligence learning models. Following that, the non-random missing values and the random missing values are handled. Randomly missing values are those that are absent from a subset of the data. Finally, the imbalanced data is balanced with complete attributes or information with the aid of random over sampler. Following preprocessing, the data is fed into the DRF feature selection algorithm, which retrieves features out of the dataset. The DBRF classification approach is used to classify the features and divide the data into attack and non-attack groups. Consequently, the DRF optimization model is used to select the most pertinent and advantageous features, hence enhancing the classifier's training speed and detection rate. The data flow is then classified as either an attacker or a normal flow based on an optimum collection of attributes using the DBRF classification model. The primary advantages of using the proposed DRF-DBRF IDS framework are increased training speed, minimal time consumption, reduced overfitting, accurate detection rate, and easy to deploy. Balanced dataset is referred to as the preprocessed or the normalized dataset that is used for subsequent intrusion detection operations. This dataset has the normalized attribute information, no missing values, and redundant information. By using the DRF algorithm, the most required subset of features are selected with its best optimum solution, which helps to train the classifier with reduced dimensionality of features. In the proposed study, there are 5 distinct and different intrusion datasets have been used for intrusion detection, and each of which having increased number of features or attributes. These are eliminated by optimally picking some selective attributes according to the best optimum solution obtained from the DRF technique. After feature reduction, the selected subset of features are passed to the DBRF classifier for training and testing operations. Based on this process, the accurate label is predicted as whether normal or attacker with high accuracy. In the proposed work, there are 5 distinct IoT intrusion datasets are used for system implementation and we are not combining these datasets together. Here, each dataset is separately used as the input for intrusion detection and classification.

### Preprocessing and normalization

The original IoT datasets are preprocessed at first for normalizing the attributes before classification, which holds the operations of NaN values handling, categorical feature extraction, and identification of missing fields. Then, it produces the balanced and normalized dataset as the output for further operations. The data is first preprocessed, which involves dealing with NAN values, categorical characteristics, unbalanced datasets, and missing values that can happen both unintentionally and purposefully. The data is then processed further afterwards this process. Preprocessing helps to gain better quality data while also lowering the challenges that come with the data, which impedes the flow of data traffic. The abbreviation NaN, which stands for "Not a Number," is one of the most frequently used symbols to denote a missing value in data when dealing with NaN numbers. The input data for an attack detection system must be free of NaN values in order to increase the accuracy of attack detection. After successfully managing NaN values, handling categorical characteristics is the next step for handling categorical features. Before categorical data is fed into the machine learning models, which is the final step, it must be processed in this stage. Machine learning models are unable to operate effectively with data that is saved in the texture format because they are regarded as mathematical models. Both randomly generated and non-randomly generated missing values are handled in the next phase of the missing value handling operation. Randomly missing values are those that are absent from certain subsamples of data. When data is absent but still has a defined structure, it's referred to as missing values. During this process, the operations such as NaN values handling, categorical attributes handling, and missing values handling at both random and not at random are performed. If the estimated ratio of both attack and non-attack samples are same, the features are directly extracted from the dataset for balancing; otherwise, the random over sampler is used to handle the imbalance information for producing the balanced dataset. The preprocessing phase handles both missing values that are not random and missing values that are missing at random. Missing values at random are those values that are absent from some subsamples of the data, which are identified when the missing data has a certain structure. Here, the NaN handling is performed to find out the missing values in the given data, which helps to increase the accuracy of intrusion detection. It is computed by using the following equation:

1 $$ DS_{N}^{{handling{ }\left( {NaN} \right)}} = {\Phi }_{{NaN_{handling} }} \left( {DS_{N} } \right) $$

where $DS_{N}$ indicates the input data, ${\Phi }_{{NaN_{handling} }}$ represents the model used to handle the NaN values, and $DS_{N}^{{handling{ }\left( {NaN} \right)}}$ indicates that is acquired after processing NaN values. Consequently, the categorical feature handling is performed NaN handling, since it is processed before being fed into the classification stage. The features are obtained by using the following models:

2 $$ DS_{N}^{{handling{ }\left( {CF} \right)}} = \varrho_{CF\_handling} \left( {DS_{N} } \right) $$

where $\varrho_{CF\_handling}$ indicates the model used to handle the categorical data, $DS_{N}^{{handling{ }\left( {CF} \right)}}$ is the output data retrieved after category processing. Moreover, the missing values are identified and handled for generating the normalized dataset. Missing values at random are those values that are absent from some subsamples of the data. Missing values—as opposed to missing data—are identified when the missing data has a certain structure. The missing values are identified by using the following equation;

3 $$ DS_{N}^{{handling\left( {Miss{ }Value} \right)}} = \delta_{{handling - missvalue{ }}}^{{\left( {R,{ }NR} \right)}} \left( {DS_{N} } \right) $$

where $\delta_{{handling - missvalue{ }}}^{{\left( {R,{ }NR} \right)}}$ represents the method used to handle the missing values, and $DS_{N}^{{handling\left( {Miss{ }Value} \right)}}$ is the output data obtained after handling missing values. Moreover, the preprocessed dataset is generated in the following form:

4 $$ DS_{N}^{PD} = \left\{ {DS_{1} ,DS_{2} ,{ }DS_{3} \ldots DS_{N} } \right\} $$

where $DS_{N}^{PD}$ denotes the preprocessed dataset, and N indicates the total number of data. The balanced and imbalanced dataset is obtained based on the ratio of attacking and non-attacking samples by using the following equation:

5 $$ DS_{N}^{PD} = \left\{ {\begin{array}{*{20}l} {DS_{N}^{B} } \hfill & {if\;\left( {X\left( {DS_{N}^{PD} } \right) = Y\left( {DS_{N}^{PD} } \right)} \right)} \hfill \\ {DS_{N}^{IB} } \hfill & {if\;\left( {X\left( {DS_{N}^{PD} } \right) \ne Y\left( {DS_{N}^{PD} } \right)} \right)} \hfill \\ \end{array} } \right. $$

where $DS_{N}^{B}$ represents the balanced dataset, $DS_{N}^{IB}$ denotes the imbalanced dataset, X and Y indicates the attacking and non-attacking data respectively. The balanced data from the collected information is added to the subsequent phase, while the imbalanced data is dealt with by a random over sampler. Here, an imbalanced dataset is handled by using a random oversampler to balance the data. By arbitrarily repeating instances from the minority class and applying them to the training input, the random oversampler creates balanced data by using the following equation:

6 $$ DS_{N}^{IB} \mathop{\longrightarrow}\limits^{Oversampling}DS_{N}^{B} . $$

Finally, the balanced dataset is obtained after oversampling, which can be used for further optimization and classification processes.

### Decisive Red Fox (DRF) optimization

After obtaining the balanced dataset from the previous stage, the DRF optimization algorithm is applied to choose the optimal features for improving the training speed and accuracy of intrusion detection. In the traditional IDS frameworks, various meta-heuristic optimization models are developed for increasing the security of networks. For instance, the Mayfly Optimization (MO), Greedy Swarm Optimization (GSO), Fruitfly Optimization (FO), and Spider Monkey Optimization (SMO) are the recently developed models used for network security. However, it has the key problems associated to the factors of complex computational operations, overfitting, reduced convergence rate, and slow in process.

Typically, the Dragon Fly Algorithm (DFA), Moth Flame Optimization (MFO), Harris Hawks Optimization (HHO), Firefly Algorithm (FA), Flower Pollination Algorithm (FPA), Whale Optimization Algorithm (WO), and Ant Lion Optimization (ALO) are some of the recently developed nature inspired/bio-inspired optimization techniques. These algorithms are extensively used in many security applications for solving the complex optimization problems. Among others, the DRF is one of the most recently developed optimization algorithm, and it has enormous benefits comparing to other techniques. It includes low computational complexity, avoids stacking of the algorithm during optimization, fast convergence, and reduced local optimum. Also, the DRF^35^ is not specifically used in the IoT-IDS security applications. Therefore, the proposed work intends to use this algorithm for optimizing the features of dataset based on the best optimal solution. Moreover, this optimization process helps to simplify the process of classification with increased attack detection rate.

This optimization algorithm can optimally tune the parameters of the balanced IoT dataset. Generally, the foxes are omnivorous, small- to medium-sized mammals that is a member of a number of Canidae genera; because of their sharp noses, thick tails, long, thin legs, and slim limbs. Also, the foxes can be differentiated from other members of their family, or giant dogs. The DRF is a new meta-heuristic optimization algorithm that draws inspiration from the red foxes' hunting habits. When hunting, the red fox approaches the target gradually while it hides in the bushes, and then the animal is suddenly attacked. This algorithm incorporates both the exploitation and exploration capabilities like other meta-heuristics models. In this algorithm, the parameter initialization is performed based on the generation of random individuals as represented in below:

7 $$ P = \left[ {p_{0} ,{ }p_{1} \ldots p_{n - 1} } \right] $$

8 $$ \left( P \right)^{i} = \left[ {\left( {p_{0} } \right)^{i} ,{ }\left( {p_{1} } \right)^{i} \ldots \left( {p_{n - 1} } \right)^{i} } \right] $$

where i indicates the number of populations in the searching space. Then, the optimum solution is achieved in the searching space by using the global optimal function. Here, the Euclidean distance is applied to obtain the optimum solution by using the following model:

9 $$ E\left( {\left( {\left( P \right)^{i} } \right)^{k} ,\left( {P_{best} } \right)^{k} } \right) = \sqrt {\left( {\left( P \right)^{i} } \right)^{k} - \left( {P_{best} } \right)^{k} } $$

where k indicates the number of iterations, $P_{best}$ is the best optimum, and $E\left( . \right)$ indicates the Euclidean distance. Consequently, the optimum solution is used to migrate all candidates as shown in below:

10 $$ \left( {\left( P \right)^{i} } \right)^{k} = \left( {\left( P \right)^{i} } \right)^{k} + rsign{ }\left( {\left( {P_{best} } \right)^{k} - \left( {\left( P \right)^{i} } \right)^{k} } \right) $$

where $r$ denotes the random number in the range of 0 to 1, which is a randomly chosen scaling hyperparameter that is set once per an iteration for the entire population. After moving to the best place, if the values of fitness at their new positions are higher, individuals stay there; otherwise, they migrate back to their original positions. This illustrates how family members return home after an expedition and teach the others where to hunt. The family members follow the explorers’ directions. If there was a chance of finding food, they would stay to hunt; otherwise, they would return home “empty-handed”. In each DRF cycle, these operations stand in for proposed global searches.

Moreover, the candidates’ new location should offer a suitable option; otherwise, the prior location would still exist. The red fox approaches the prey to observe it, which is characterized as the use of the DRF modelled by assuming a random number $\omega$ between [0, 1]:

11 $$ \left\{ {\begin{array}{*{20}l} {Move\;forward} \hfill & {if,\omega > 3/4{ }} \hfill \\ {Stay\;hidden} \hfill & {if,\omega > 3/4} \hfill \\ \end{array} } \right. $$

12 $$ \omega = \left\{ {\begin{array}{*{20}l} {h \times \frac{{{\text{sin}}\left( {\delta_{0} } \right)}}{{\delta_{0} }}} \hfill & {if\;\delta_{0} \ne 0} \hfill \\ \tau \hfill & {if\;\delta_{0} = 0} \hfill \\ \end{array} } \right. $$

where h is the random number in the range of [0, 0.2], $\delta_{0}$ is also a random number lies in the range of [0, 2 $\pi$] that is considered as the fox observation angle, and $\tau$ denotes the random value in the range of 0 to 1. The following system of equations for spatial coordinates are used to model motions for the population of individuals.

13 $$ \left\{ {\begin{array}{*{20}l} {p_{0}^{new} = h \times \omega \times \cos \left( {\delta_{1} } \right) + p_{0}^{actual} } \hfill \\ {p_{1}^{new} = h \times \omega \times \sin \left( {\delta_{1} } \right) + h \times \omega \times \cos \left( {\delta_{2} } \right) + p_{1}^{actual} } \hfill \\ {p_{1}^{new} = h \times \omega \times \sin \left( {\delta_{1} } \right) + h \times \omega \times \sin \left( {\delta_{2} } \right) + h \times \omega \times \cos \left( {\delta_{3} } \right) + p_{2}^{actual} } \hfill \\ \vdots \hfill \\ {p_{n - 1}^{new} = h \times \omega \times \mathop \sum \limits_{t = 1}^{n - 2} \sin \left( {\delta_{1} } \right) + h \times \omega \times \cos \left( {\delta_{n - 1} } \right) + p_{n - 2}^{actual} } \hfill \\ {p_{n - 1}^{new} = h \times \omega \times \sin \left( {\delta_{1} } \right) + h \times \omega \times \sin \left( {\delta_{2} } \right) + \ldots + h \times \omega \times \sin \left( {\delta_{n - 1} } \right) + p_{n - a}^{actual} } \hfill \\ \end{array} } \right. $$

In order to maintain a fixed size of the population, the population's worst members were eliminated, and many new members were added. Subsequently, two optimal members are identified at iteration k, and their center is estimated as follows:

14 $$ C_{e}^{k} = \frac{1}{2}\left( {P\left( 1 \right)} \right)^{k} - \left( {P\left( 2 \right)} \right)^{k} $$

here a random parameter $\varphi$ between (0 and 1) is used for each iteration that specifies replacements in the iteration in accordance with the following model:

15 $$ \left\{ {\begin{array}{*{20}l} {new\;nomadic\;individual} \hfill & {if,\;\varphi > 0.45} \hfill \\ {reproduction} \hfill & {if,\;\varphi \le 0.45} \hfill \\ \end{array} } \right. $$

Based on this process, the random locations are updated in the searching space, and the new members are added by using the following model:

16 $$ \left( {P^{rp} } \right)^{k} = \frac{\varphi }{2}\left( {P\left( 1 \right)} \right)^{k} - \left( {P\left( 2 \right)} \right)^{k} $$

By using this function, the reproduced individual is obtained, and the best $P_{best}$ is returned as the output. This function can be used to optimally select the features for training the data samples of the classifier.

### Descriptive back propagated: radial basis function (DBRF) network classification

After feature optimization, the DBRF network classification model is implemented to categorize the data flow as whether normal or intrusion. In the traditional works, various machine learning and deep learning based classification techniques are implemented to increase the security of IoT networks by protecting it from the harmful intrusions. For instance, the Logistic Regression (LR), Decision Tree (DT), eXtreme Gradient Boost (XGB), Convolutional Neural Network (CNN), and ensemble learning models are extensively used in many network security applications. However, it has the major problems of inaccurate prediction if the sample is too sample, overlapping, higher training time, and unstability^36–38^. Therefore, the proposed work motivates to develop a new classification model, named as, DBRF for increasing the security of IoT networks. The proposed DBRF^39^ provides enormous benefits such as simple design, high adaptation, great input noise tolerance, and online learning capability. Also, a robust networking systems can be designed extremely well owing to the characteristics of DBRF networks. It is a kind of learning model that distributes the input space among local kernels. A portion of these locally tailored kernel units are engaged for each input data point, depending on where in the input space it appears. It appears as though these local units have assigned each of them a portion of the input area to manage. The concept of locality itself suggests the requirement for a distance function that gauges how similar provided input data with dimensionality is to the center of each kernel unit. The Euclidean distance is computed between the input data and center for estimating the response function of the classifier. The concept behind employing such local models is that we define a basis function for each of these clusters if we presume that there are groups of data points in the training data. According to the non-linearity function, the DBRF can accurately predict the data into the corresponding class. Moreover, the hyperbolic function and error function are computed in this model during the training phase.

Due to the intrinsic ability of the radial basis function network model to learn the underlying distribution of training data, the DBRF classifier is employed here. In this model, the Gaussian function $G_{f}$ is estimated by using the input data and its center as shown in below:

17 $$ G_{f} = exp\left[ { - \frac{{\left| {\left| {D - q_{x} } \right|} \right|^{2} }}{{2\sigma^{2} }}} \right] $$

where D indicates the input data, $q_{x}$ is the center of kernel unit, and $\sigma$ denotes the standard deviation. Following the discovery of these cluster centers and spreads, the output of the response function is considered as the input to a perceptron as shown in below:

18 $$ b = f\left( {\mathop \sum \limits_{x = 1}^{X} \omega_{x} G_{f} + \omega_{0} } \right) $$

where $f\left( . \right)$ denotes the non-linearity function, X indicates the number of basis functions, $\omega_{x}$ represents the weight value associated to the unit x, and $\omega_{0}$ is the bias value. After that, the hyperbolic tanh function is applied to reduce the error rate at the time of training. Then, the function is computed as follows:

19 $$ \varepsilon = \frac{1}{2}\left( {k - b} \right)^{2} $$

20 $$ b = \tanh \left( m \right) $$

21 $$ m = \mathop \sum \limits_{x = 1}^{X} \omega_{x} G_{f} + \omega_{0} . $$

Consequently, the learning rate rule updation is performed, and the output class label is predicted as shown in below:

22 $$ OC\left( Y \right) = \left\{ {\begin{array}{*{20}l} {Normal} \hfill & {if,b \ge \left( {\overline{b} - \sigma_{B} } \right)} \hfill \\ {Intrusion} \hfill & {if,b < (\overline{b} - \sigma_{B} } \hfill \\ \end{array} } \right.. $$

By using this model, the normal and intrusion classes are accurately predicted from the given IoT datasets. The primary benefits of using the proposed DRF-DBRF IoT security framework are as follows:

- Increased speed of training
- Accurate intrusion detection rate
- Easy to implement and understand
- Computational efficient
- Reduced overall time consumption

## Results

This section validates the performance and results of the proposed DRF-DBRF security model by using various evaluation parameters. In this system, the most popular and different IoT benchmarking datasets are used to validate the system, which includes IoTID-20, NetFlow-IoT-v2, ToN-IoT, NSL-KDD, UNSW-NB 15. Moreover, the obtained results are compared with some of the baseline IoT IDS security frameworks for proving the superiority of the proposed model. The parameters used to assess the results are computed by using the following equations:

23 $$ Accuracy = { }\frac{TrP + TrN}{{TrP + TrN + FaP + FaN}} \times 100{\text{\% }} $$

24 $$ Precision = { }\frac{TrP}{{TrP + FaP}} \times 100{\text{\% }} $$

25 $$ F1{\text{-}}score = { }\frac{2 \times Pre \times Sen}{{Pre + Sen}} \times 100{\text{\% }} $$

26 $$ Recall = { }\frac{TrP}{{TrP + FaN}} \times 100{\text{\% }} $$

27 $$ Sensitivity = { }\frac{TrP}{{TrP + FaN}} \times 100{\text{\% }} $$

28 $$ Specificity = { }\frac{TrN}{{TrN + FaP}} \times 100{\text{\% }} $$

where TrP—true positive, TrN—true negative, FaP—false positive, and FaN—false negative. The list of datasets used to validate the system model are presented in Table 1.

**Table 1:** List of IoT datasets used in this study.

Datasets	Description
Dataset 1	IoTID20
Dataset 2	NetFlow-IoT-v2,
Dataset 3	Netflow-ToN-IoT
Dataset 4	NSL-KDD
Dataset 5	UNSW-NB 15

The dataset descriptions are provided for all these datasets with the number of samples and attacking classes in Tables 2, 3, 4, 5 and 6. These IoT datasets are extensively used in many network application systems for increasing the security of IoT networks. To assess the overall performance and intrusion detection efficiency of the proposed DRF-DBRF security model, these 6 different types of IoT datasets have been used in this work.

**Table 2:** Dataset description of IoTID20.

Label name	Value	No of samples
Label	Normal	13,859
	Anomaly	586,241
Attack	Normal	40,073
	DoS	59,391
	Mirai	415,677
	MITM ARP	35,377
	Spoofing	75,265
	Scan

**Table 3:** Dataset description of Netflow-ToN-IoT.

Label name	Value	No of samples
Label	Normal	270,279
	Anomaly	1,379,274
Attack	Ransomware	142
	Benign	270,279
	XSS	99,944
	Scanning	21,467
	Password	156,299
	DoS	17,717
	DDoS	326,345
	Injection	468,539
	MITM	1295

**Table 4:** Dataset description of Netflow-BoT-IoT.

Label name	Value	No of samples
Label	Normal	13,859
	Anomaly	586,241
Attack	Benign	13,859
	Reconaissance	470,655
	DDoS	56,844
	DoS	56,833
	Theft	1909

**Table 5:** Dataset description for UNSW-NB-15 dataset.

Type	Training	Testing
Worms	130	44
Shellcode	1133	378
Backdoor	1746	583
Analysis	2000	677
Reconnaissance	10,491	3496
DoS	12,264	4089
Fuzzers	18,184	6062
Exploits	33,393	11,132
Generic	40,000	18,871
Normal	56,000	37,000

**Table 6:** Dataset description for NSL-KDD.

Category	NSL-KDD training	NSL-KDD testing
Total no of instances	125,973	22,544
Normal	67,343	9711
DoS	45,927	7460
U2R	52	67
R2L	995	2885
Probe	11,656	2421

**Figure 3:**
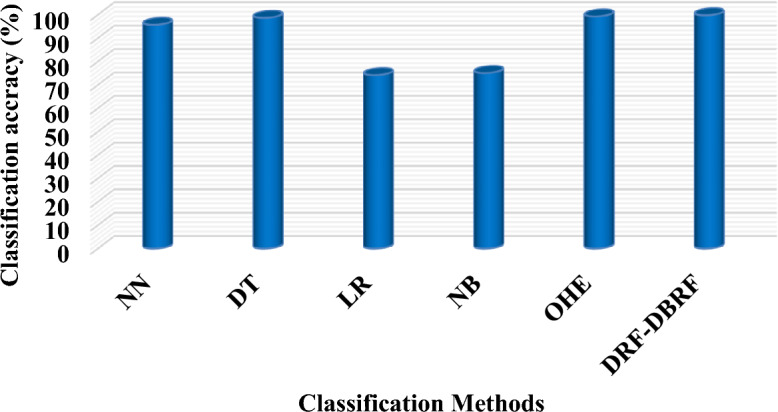
Classification accuracy using IoTID-20 dataset.

**Figure 4:**
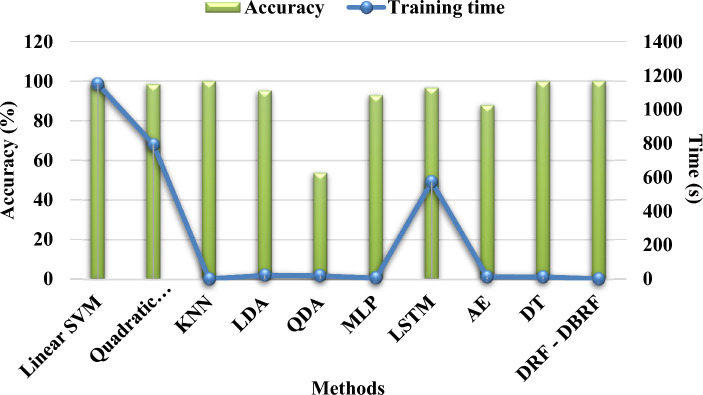
Comparative analysis based on training time and accuracy using IoT-ID20 dataset.

Table 7 and Fig. 3 compares the classification accuracy of the traditional and proposed AI based detection methodologies used for IoT security by using IoT-IDS20 dataset^40^. Typically, the classification accuracy is one of the most prominent measure used to determine the overall intrusion detection rate of IDS framework. Consequently, the training time and accuracy (%) of the existing and proposed intrusion detection mechanisms are validated by using the IoT-IDS 20 dataset as shown in Table 8 and Fig. 4. In general, the increased classifier’s training time indicates the reduced performance of the detection system. Hence, the training time of classifier must be reduced to the maximum. According to the results, it is analyzed that the proposed DRF-DBRF technique outperforms the other classification approaches with increased classification accuracy and training time. Due to the utilization of DRF algorithm, the training speed and effectiveness of the classifier is highly improved.

**Table 7:** Classification accuracy of IoT-ID20 dataset.

Methods	Classification accuracy (%)
Neural Network (NN)	95.70
Decision Tree (DT)	98.70
Logistic Regression (LR)	74.20
Naïve Bayes (NB)	75.10
One hot encoding model—RF	99.3
Proposed DRF-DBRF	99.8

**Table 8:** Training time and accuracy analysis using IoT-ID20 dataset.

Methods	Training time (μs/flow)	Training accuracy (%)
Linear SVM	1150.57	98.16
Quadratic SVM	792.61	98.25
KNN	0.17	99.79
LDA	21.6	95.07
QDA	18.89	53.6
MLP	4.94	92.71
LSTM	572.44	96.53
AE	13.35	87.74
DT	12.49	99.69
Proposed DRF—DBRF	0.12	99.9

Table 7 compares the classification accuracy of the proposed DRF-DSRF mechanisms with that of the traditional NN, DT, LR, NB, and one-hot encoding models using the IoT-ID 20 dataset. Using data samples from the IoT-ID20 dataset, this assessment compares some of the most popular and widely utilized machine learning approaches with the proposed DRF-DBRF model. One of the most crucial and crucial parameters used to verify the attack detection effectiveness of the classifier is classification accuracy. The suggested DRF-DBRF model surpasses the other current machine learning algorithms with higher classification accuracy, as shown by the prediction results. The primary factor in the proposed framework's enhanced performance is the use of the DRF optimization technique, which lowers the dimensionality of features prior to classification.

Similar to this, Table 8 compares and validates the training times and training accuracy of the proposed and standard models. Techniques including linear SVM, quadratic SVM, LDA, KNN, QDA, MLP, LSTM, AE, and DT are taken into account for this comparative analysis. In this work, the aforementioned classification algorithms are contrasted in order to assess the training performance of the suggested DBRF classification strategy. Final results show that the suggested DBRF outperforms traditional methods with higher training accuracy and shorter training times. The suggested DRF optimization technique extracts the pertinent subset of features from the provided intrusion dataset, speeding up the classifier's training process and reducing the overall training time with high accuracy.

**Figure 5:**
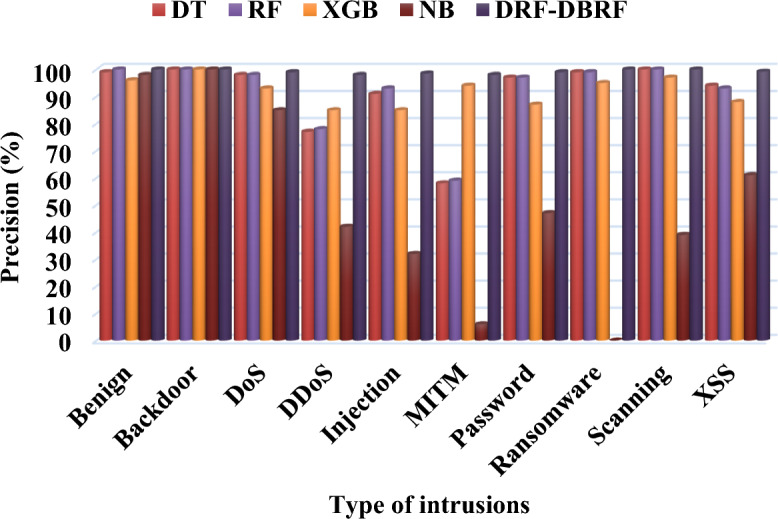
Precision analysis using NetFlow-BoT-IoT-v2.

**Figure 6:**
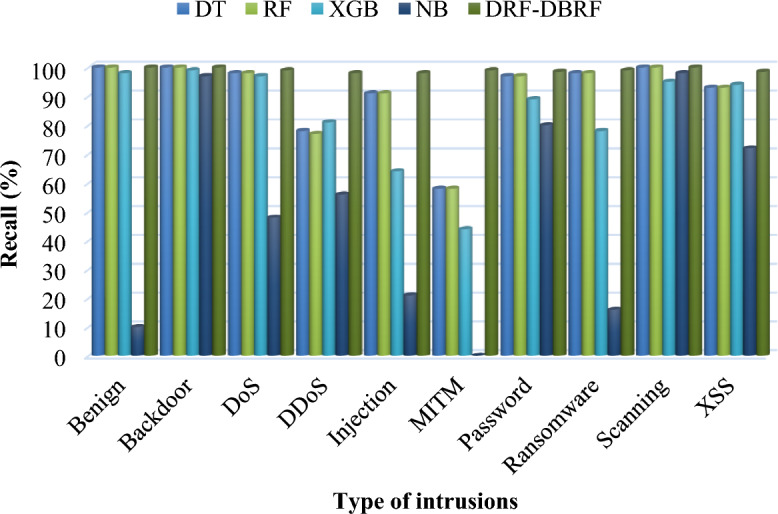
Comparative analysis based on recall using NetFlow-BoT-IoT-v2.

**Figure 7:**
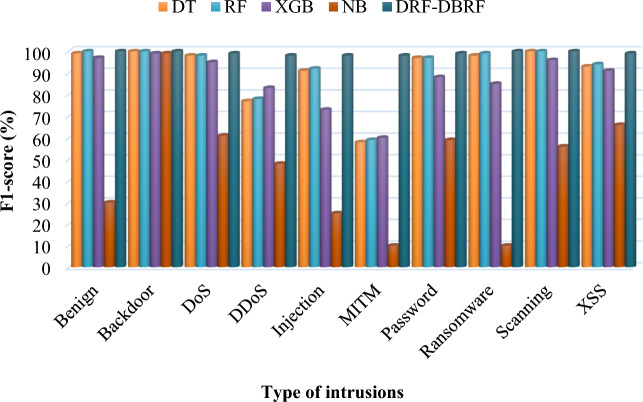
Comparative analysis based on f1-score using NetFlow-BoT-IoT-v2.

Table 9 and Fig. 5 compares the precision conventional^41^ and proposed security methodologies by using Netflow-BoT-IoT-v2 dataset. Typically, precision is the metric that assesses a performance of the model by determining how frequently the model's forecast is accurate when it correctly foresees an occurrence. Consequently, Tables 10 and 11 compares the recall and f-measure values of existing and proposed IDS methodologies by using NetFlow-BoT-IoT-v2 dataset. Then, its corresponding graphical illustrations are represented in Figs. 6 and 7. The recall is also termed as detection rate/true positive rate, which is an indicator of how well the machine learning model detected the occurrences of True Positives. Moreover, it validates that how well the model recognizes pertinent facts. Moreover, the total accuracy of classifier is determined based on the trade-off between recall and precision, which considers both false positives and false negatives. According to the improved values of these parameters, the overall detection efficacy of the classifier is determined. For class-wise evaluation of the classifier's output, these criteria are helpful. The harmonic mean of recall and precision is the F score, if the maximum value of 1, which denotes the perfect precision and recall, and a minimum value of 0 can occur when either precision or recall is zero. The F score is also more useful criteria than accuracy in classes with unequal distribution. Based on the overall analysis, it is observed that the proposed DRF-DBRF outperforms the other approaches with increased precision, recall, and f1-score values. Due to the proper dataset balancing and attributes tuning, the training of classifier is highly improved, which helps to improve these parameters.

**Table 9:** Precision using NetFlow-BoT-IoT-v2.

Classes	DT	RF	XGB	NB	DRF-DBRF
Benign	99	100	96	98	100
Backdoor	100	100	100	100	100
DoS	98	98	93	85	99
DDoS	77	78	85	42	98
Injection	91	93	85	32	98.5
MITM	58	59	94	6	98
Password	97	97	87	47	99
Ransomware	99	99	95	0	100
Scanning	100	100	97	39	100
XSS	94	93	88	61	99.2

**Table 10:** Recall using NetFlow-BoT-IoT-v2.

Classes	DT	RF	XGB	NB	DRF-DBRF
Benign	100	100	98	10	100
Backdoor	100	100	99	97	100
DoS	98	98	97	48	99
DDoS	78	77	81	56	98
Injection	91	91	64	21	98
MITM	58	58	44	0	99
Password	97	97	89	80	98.5
Ransomware	98	98	78	16	99
Scanning	100	100	95	98	100
XSS	93	93	94	72	98.5

**Table 11:** F1-score using NetFlow-BoT-IoT-v2.

Classes	DT	RF	XGB	NB	DRF-DBRF
Benign	99	100	97	30	100
Backdoor	100	100	99	99	100
DoS	98	98	95	61	99
DDoS	77	78	83	48	98
Injection	91	92	73	25	98
MITM	58	59	60	10	98
Password	97	97	88	59	99
Ransomware	98	99	85	10	100
Scanning	100	100	96	56	100
XSS	93	94	91	66	99

**Figure 8:**
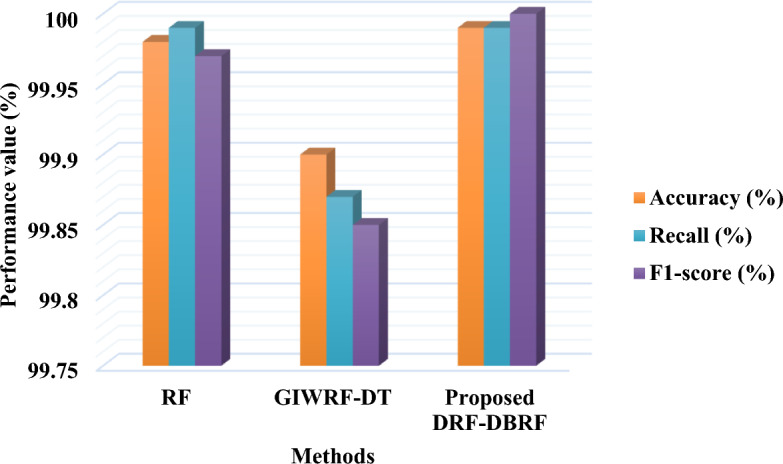
Performance evaluation using ToN-IoT dataset.

Figure 8 and Table 12 compares the accuracy, recall, and f1-score of the existing and proposed classification methodologies by using ToN-IoT dataset. In Table 12, the conventional approaches including, Gini Impurity based Weighted Random Forest (GIWRF) integrated with Decision Tree (DT) and Random Forest (RF) are considered into account for comparison. In order to analyze the intrusion detection efficacy and competence of the proposed security model, various IoT datasets are considered in this study during evaluation. The observed results also indicate that the combination of DRF-DBRF outperforms the other existing models with increased accuracy, recall, and f1-score values.

**Table 12:** Comparative analysis based on ToN-IoT dataset.

Methods	Accuracy (%)	Recall (%)	F1-score (%)
RF	99.98	99.99	99.97
GIWRF-DT	99.90	99.87	99.85
Proposed DRF-DBRF	99.99	99.99	100

**Figure 9:**
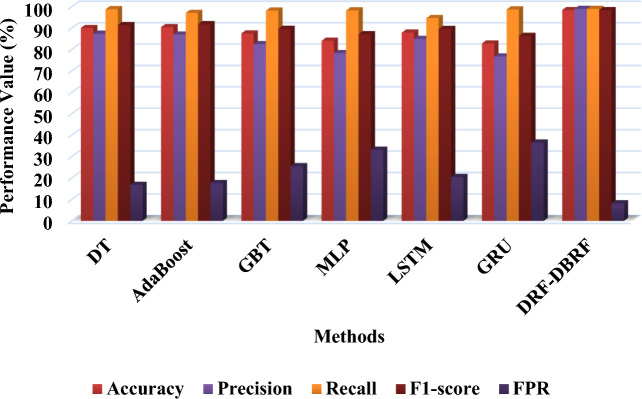
Overall performance analysis based on UNSW-NB15 dataset.

**Figure 10:**
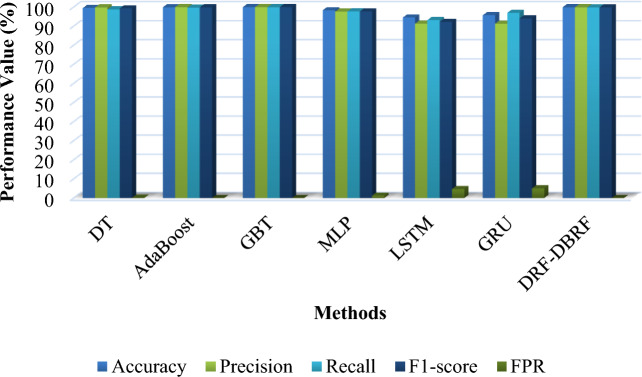
Overall performance analysis based on ToN-IoT dataset.

Tables 13 and 14 presents the overall comparative analysis of the existing^42^ and proposed anomaly detection methodologies by using the UNSW-NB 15 and ToN-IoT datasets respectively. Then, its corresponding graphical evaluations are depicted in Figs. 9 and 10. The proposed solution once more outperforms the other methods in terms of high performance values. The methods are compared in this experiment based on how well they can predict the actual classes of dataset records. The overall performance findings on this dataset further support the proposed approach's superiority over previous classification approaches.

**Table 13:** Overall comparative analysis using UNSW-NB 15 dataset.

Techniques	Accuracy	Precision	Recall	F1-score	FPR
DT	90.15	87.45	98.85	91.46	16.84
AdaBoost	90.51	87.07	97.19	91.85	17.67
GBT	87.56	82.49	98.25	89.68	25.54
MLP	84.11	78.34	98.31	87.20	33.28
LSTM	87.90	85.01	94.71	89.60	20.44
GRU	82.87	76.78	98.75	86.39	36.57
DRF-DBRF	98.5	99	99	98.5	8.2

**Table 14:** Overall comparative analysis using ToN-IoT dataset.

Techniques	Accuracy	Precision	Recall	F1-score	FPR
DT	99.50	99.83	98.74	99.28	0.09
AdaBoost	99.88	99.99	99.67	99.83	0.001
GBT	99.98	99.98	99.95	99.97	0.006
MLP	98.35	97.60	97.68	97.64	1.2
LSTM	94.51	91.28	93.18	92.22	4.7
GRU	95.69	91.23	96.99	94.02	5.0
DRF-DBRF	99.9	99.9	99.8	99.8	0.001

**Figure 11:**
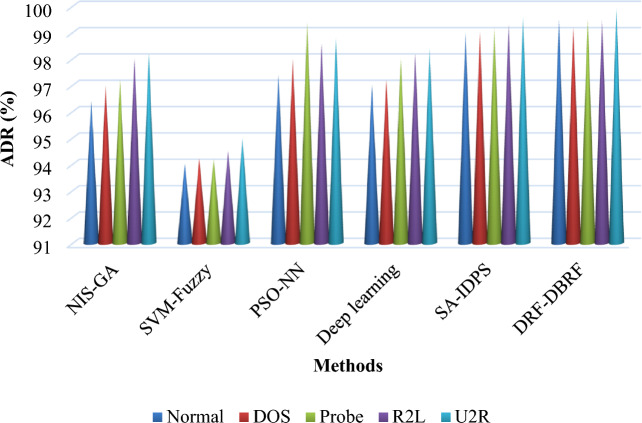
Attack detection rate using NSL-KDD dataset.

Table 15 and Fig. 11 compares the Attack Detection Rate (ADR) of previous and proposed anomaly detection methodologies by using the NSL-KDD dataset. In comparison to the existing study, models created using the proposed approach are more capable of effectively classifying the legitimate and malicious data flows from the given IoT datasets.

**Table 15:** Comparative analysis of ADR between existing and proposed IDS frameworks.

Methods	Normal	DOS	Probe	R2L	U2R
NIS-GA	96.4	97	97.2	98	98.2
SVM-fuzzy	94	94.2	94.2	94.5	95
PSO-NN	97.4	98	99.4	98.6	98.8
Deep learning	97	97.2	98	98.2	98.4
SA-IDPS	99	99	99.2	99.3	99.6
DRF-DBRF	99.5	99.2	99.5	99.5	99.9

Typically, the computational complexity is one of the most essential parameter used to assess the overall efficacy of the algorithm. Moreover, the computational cost of optimization can be computed according to the parameters of population size, dimension of the problem, and number of iterations required to reach the optimal solution. In this security framework, the proposed DRF mechanism requires the maximum of 100 number of iterations with the population size of 30. Table 16 validates the computational time of the existing^15^ and proposed optimization based IDS methodologies using different IoT-IDS datasets. The existing techniques considered in this evaluation are Harris Hawks Optimization (HHO), Gorilla Troop Optimizer-Binary Swarm Algorithm (GTO-BSA), and Hunger Game Search (HGS). The results demonstrates that the proposed DBRF classifier outperforms the other classifiers with the inclusion of DRF optimization algorithm. In addition, Table 17 validates and compares the accuracy of some of the most extensively classification approaches in the intrusion detection systems with the proposed DRF-DBRF approach. For this assessment, all of five datasets used in this study are considered into account for comparison. The findings state that the proposed DRF-DBRF outperforms the other classification approaches with increased accuracy.

**Table 16:** Computational time analysis.

Datasets	Parameters	HHO	GTO-BSA	HGS	Proposed
NSL-KDD	Mean	12,476.16	10,205.83	661.72	554.23
	STD	2498.787	1531.406	325.91	229.48
BoT-IoT	Mean	146.24	145.74	12.57	10.32
	STD	18.83	4.53	10.47	8.46
UNSW-NB 15	Mean	144.71	161.23	10.74	8.58
	STD	5.98	4.89	8.57	6.47

**Table 17:** Accuracy of conventional and proposed classifiers using all intrusion datasets.

Datasets	Accuracy (%)				
	LR	DT	RF	LSTM	Proposed
IoTID20	90	95	94	97	99.3
NetFlow-IoT-v2	91	96	95	97.5	99.4
NetFlow-ToN-IoT	89	94	93	96	99
NSL-KDD	90.8	92	94.2	98.4	99.5
UNSW-NB 15	92	93.1	95.4	98.9	99.4

## Conclusion

This paper presents an enhanced DRF-DBRF classification model addressing the intrusion detection problems in the IoT systems. Initially, the data normalization is performed with the operations of NaN values handling, categorical feature extraction, and missing field identification. The NaN processing is carried out in this case to identify the missing values in the supplied data, which contributes to improving the precision of intrusion detection. Since the categorical feature is processed before being fed into the classification stage, the handling of the categorical feature is conducted NaN handling. Additionally, the missing values are located and dealt with in order to create the standardized dataset. Here, the DRF optimization approach is used to extract the pertinent features from the balanced IoT datasets, which speeds up the classifier's training process. When compared to the optimization techniques, the primary reasons of using the DRF algorithms are as follows: increased convergence rate, training speed, and reduced overfitting. Based on the optimized characteristics, the DBRF classification process is used to identify and classify the type of intrusions. The radial basis function network model’*s inherent capacity to understand the underlying distribution of training data is the reason the DBRF classifier is used in this context. The normal and intrusion classes are correctly predicted from the provided IoT datasets based on the learning rule update. Moreover, the performance of the proposed DRF-DBRF model is validated and tested by using five different datasets, and the estimated results are compared with the recent anomaly detection approaches. From the overall observed results, it is analyzed that the combination of DRF-DBRF overwhelms the other anomaly detection techniques with increased precision (99%), accuracy (99.2%), recall (99%), and f1-score (98.9%). Moreover, the results are highly superior to the existing techniques, which shows the improved performance and competence of the proposed model.

In future, the present work can be extended by implementing the IDS framework to the IoT integrated smart application systems.

## Data Availability

The data that support the findings of this study are available from the corresponding author, upon reasonable request.
